# Assessment of the Radioactivity, Metals Content and Mineralogy of Granodiorite from Calabria, Southern Italy: A Case Study

**DOI:** 10.3390/ma17153813

**Published:** 2024-08-02

**Authors:** Luigi Dattola, Alberto Belvedere, Maurizio D’Agostino, Giuliana Faggio, Domenico Majolino, Santina Marguccio, Giacomo Messina, Maurizio Messina, Antonio Francesco Mottese, Giuseppe Paladini, Valentina Venuti, Francesco Caridi

**Affiliations:** 1 Agenzia Regionale per la Protezione dell’Ambiente della Calabria (ARPACal)—Centro Regionale Geologia e Amianto, Via della Pace, 87050 Castrolibero, Italy; l.dattola@arpacal.it; 2 Dipartimento di Ingegneria dell’Ambiente (DIAm), Università della Calabria, Via Pietro Bucci, 87036 Rende, Italy; 3 Agenzia Regionale per la Protezione dell’Ambiente della Calabria (ARPACal)—Dipartimento di Reggio Calabria, Via Troncovito SNC, 89135 Reggio Calabria, Italy; a.belvedere@arpacal.it (A.B.); m.dagostino@arpacal.it (M.D.); s.marguccio@arpacal.it (S.M.); m.messina@arpacal.it (M.M.); 4 Dipartimento di Ingegneria dell’Informazione, delle Infrastrutture e dell’Energia Sostenibile (DIIES), Università “Mediterranea”, Loc. Feo di Vito, 89122 Reggio Calabria, Italy; gfaggio@unirc.it (G.F.); messina@unirc.it (G.M.); antonio.mottese@unirc.it (A.F.M.); 5 Dipartimento di Scienze Matematiche e Informatiche, Scienze Fisiche e Scienze della Terra, Università degli Studi di Messina, V.le F. Stagno D’Alcontres, 31, 98166 Messina, Italy; dmajolino@unime.it (D.M.); gpaladini@unime.it (G.P.); vvenuti@unime.it (V.V.)

**Keywords:** granodiorite, radioactivity, radiological risk, radon exhalation, metals, pollution, mineralogy

## Abstract

In this paper, an assessment of the natural radioactivity level, radon exhalation, metal contamination, and mineralogy of a granodiorite rock sample from Stilo, in the Calabria region, Southern Italy is presented as a case study. This rock was employed as a building material in the area under study. The specific activity of ^226^Ra, ^232^Th and ^40^K natural radioisotopes was assessed through high-purity germanium (HPGe) gamma-ray spectrometry. Then, several indices such as the absorbed gamma dose rate (D), the annual effective dose equivalent (AEDE), the activity concentration index (ACI) and the alpha index (I_α_), were quantified to determine any potential radiological health risk related to radiation exposure from the analyzed rock. Furthermore, E-PERM electret ion chambers and inductively coupled plasma mass spectrometry (ICP-MS) measurements were carried out to properly quantify the radon exhalation rate and any possible metal pollution, respectively. In particular, to further address metal pollution factors, the geo-accumulation index (I_geo_) was calculated to properly address the toxicity levels of the ecosystem originating from the detected metals. Finally, with the aim of successfully discriminating the provenance of such naturally occurring radionuclides, a combined approach involving X-ray diffraction (XRD) and µ-Raman spectroscopy was employed for the identification of the main radioisotope-bearing minerals characterizing the investigated granodiorite. The results achieved in this case study can be taken as the basis for further inquiries into background levels of radioactivity and chemical contamination in natural stone employed as building materials.

## 1. Introduction

Environmental natural radioactivity represents a major contribution to a population’s exposure to ionizing radiation, originating from both cosmogenic radionuclides and primordial radioisotopes existing in the upper rigid part of the lithosphere (the Earth’s crust) [1]. From the standpoint of natural radioactivity, nuclides belonging to the radioactive chains of ^238^U, ^232^Th, ^235^U, and primordial ^40^K, are noteworthy [2]. These naturally occurring radioisotopes can generally be encountered in environmental matrices of different typologies, especially in soil, water and rocks [3], the latter often being employed as building materials [4]. Therefore, studies aimed at evaluating the content of natural radioactivity in rocks provide the essential tools for radiological risk assessment related to human beings upon exposure to ionizing radiation [5]. This task turns out to be crucial since, according to the literature [6], long-term exposure to uranium, radium and thorium through ingestion or inhalation can cause a number of health effects [7]. In addition, the risk of lung cancer may rise with prolonged exposure to radon gas (^222^Rn) [8]. In fact, the World Health Organization (WHO) has identified radon and its progeny as the second largest risk factor for developing lung cancer, after smoking, in the general population [9]. It has been determined that internal exposure to radon progeny accounts for almost half of the average annual effective dose of radioactivity that humans receive from natural sources worldwide [10]. Thus, in order to determine the radiological risk to human health, that increases in smokers and is mainly due to ^214^Po and ^218^Po radioisotopes, it is essential to monitor the concentrations of radon and its products in the environment [11]. The main source of indoor radon at the ground and basement level is the pressure-driven influx from subsurface soil, even though building materials with high concentrations of ^226^Ra may represent another significant source of natural radiation [12], i.e., the only one for higher floors, taking into account that ^226^Ra decays to ^222^Rn, in the ^238^U decay chain. Different types of rocks contain traces of uranium and/or radium, ranging from between 2 and 5 mg kg^−1^ (background) to 1000 mg kg^−1^ (black shales) [13]. In the case of igneous rocks, specifically, crystalline rocks such as granites, granitic pegmatites, and syenites are more likely to contain uranium [14]. Most of the uranium/radium concentration in these rocks is found in accessory minerals like zircon, monazite, allanite, and sphene [15]. The exhalation of ^222^Rn from a given rock surface is strongly influenced by the petrographic and petrophysical characteristics of the rock itself (i.e., micro-fissures, grain size, arrangement, alteration degree, and contact surfaces between constituents), even though measurements of U/Ra activity can provide useful information about radon release potential [16]. Therefore, to determine the actual risk associated with a particular natural stone, measurements of ^222^Rn exhalation rates must be carried out in real-time [17].

Furthermore, as a result of unchecked urbanization and rapid industrialization, an alarming number of chemical contaminants are currently prevalent in many cities and locations [18]. Metals are among the pollutants of greatest concern due to their bioaccumulative and persistent nature [19]. In addressing the quantity of pollution itself, a knowledge of the metals’ provenance and contamination mechanisms turns out to be fundamental, especially in materials/environments characterized by concentration levels close to the toxicity threshold [20]. Given the harmful consequences that metal pollution has on living beings when the permitted concentration thresholds are exceeded, environmental contamination by metals is a global issue and must therefore be deeply investigated [21,22,23].

Finally, geochemical studies of rocks can clarify patterns of element distribution, in turn related to the existing environmental conditions of a given area [24].

In the present study, a multidisciplinary approach involving high purity germanium (HPGe) gamma-ray spectrometry, inductively coupled plasma mass spectrometry (ICP-MS), X-ray diffractometry (XRD) and µ-Raman spectroscopy was employed to assess the radioactivity and metal content of a granodiorite sample from Stilo, Calabria region, Southern Italy, and to properly correlate the observed radioactivity content with both the mineralogical and geochemical composition of the investigated specimen. In addition, the absorbed gamma dose rate (D), the annual effective dose equivalent (AEDE), the activity concentration index (ACI) and the alpha index (I_α_) were calculated to estimate the potential radiological hazard related to radiation exposure from the analyzed rock. Lastly, the degree of harmfulness that metals impose on the ecosystem, the geo-accumulation index (I_geo_), was also computed. It is worthy of note that the analyzed sample was largely employed as a building material in the area under study, and external paving elements, slabs, portal elements as well as other artifacts can still be seen in the quarry service yard.

## 2. Geological Setting

The Calabria-Peloritani Orogen is a system consisting of tectonic units that were stacked up during the Alpine Orogenesis and distributed along the entire Calabrian region and part of Northern Sicily. During the various orogenetic phases, and until the conclusion of the Alpine Orogenesis, there have been migrations that led this system to occupy the current geographical position with, in addition, a sedimentary cover that began to form in the Mesozoic Era and continues until the present day [25,26,27,28].

The study area is located in the central portion of the Calabria-Peloritani Orogen, between the Curinga-Girifalco line to the north and the Palmi-Antonimina line to the south, at the edge of the Variscan crystalline basement that constitutes the Serre Massif.

In that area, the Mammola Complex and the Stilo-Pazzano Complex crop out, separated by a low-angle tectonic contact. Both complexes show a contact metamorphism imprint due to the intrusion of the Late Variscan granitoids. Above the Stilo-Pazzano Complex lies, transgressive and discordant, a meso-cenozoic sedimentary succession.

This succession begins with Mesozoic dolomites and limestones passing, by a discordant surface, to rocks belonging to the Stilo-Capo d’Orlando Formation, which was deposited between the late Oligocene and early Miocene. The Stilo-Capo d’Orlando Formation emerges in the southern sector of Calabria and, in the study area, consists largely of polygenic conglomerates, whose clasts are predominantly granitoid elements and, subordinately, schists and dolomitic limestone elements.

The conglomerate, with irregular stratification, lies south of mount Consolino, mount Stella and mount Gallo, between the Stilaro river and the Precariti valley [26,29]. Then, from the oldest terms to the younger ones, the Variegated Clays, a succession that sedimented between the Serravallian and Tortonian age, consisting mainly of conglomerates containing large blocks of granitoid rocks and coarse sands, and the Trubi Formation.

Going on, the geological setting of the Calabria-Peloritani Orogen suggests that naturally occurring radionuclides may be present in significant concentrations. The granitoid intrusions, which are typical of Late Variscan magmatic activities, are often associated with higher concentrations of uranium and thorium. Uranium formation in this context could be attributed to magmatic fractionation processes during the crystallization of granitoid magmas. Additionally, hydrothermal processes might contribute to the mobilization and concentration of uranium, thorium, and other radionuclides within the geological structures. Noteworthy, regions with similar granitoid intrusions often exhibit higher background levels of natural radionuclides [30,31]. Hence, it is plausible to infer that the study area might also possess high contents of natural radionuclides, warranting further investigation to quantify these concentrations.

## 3. Materials and Methods

### 3.1. Sampling

The granodiorite sample was taken from a quarry, now closed, located along Provincial Road No. 9 which leads from Monasterace Marina to Stilo. Five aliquots of the granodiorite were collected, in accordance with [32] and then carefully stocked up in labeled plastic containers to avoid contamination. GPS coordinates of the sampling site are as follows: 38°27′53.10″ N, 16°29′24.67″ E.

**Figure 1:**
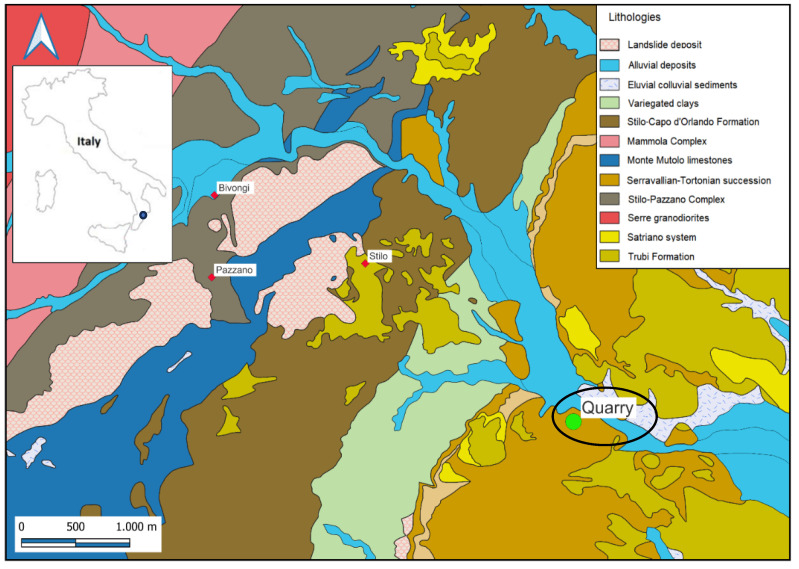
The geological sketch map of the investigated area, with the quarry site marked.

The geological map of the investigated area, together with the position of the quarry site, are shown in Figure 1.

### 3.2. HPGe Gamma Spectrometry

For the gamma spectrometry measurements, each aliquot of the analyzed sample was dried at 105 °C in an oven, powdered in a vibratory micro mill, sieved to obtain a particle size less than 2 mm, then placed in a Marinelli hermetically sealed container of 250 mL capacity and left to stand for a period of 30 days in order to reach the secular radioactive equilibrium between ^226^Ra and its short-lived gamma daughters [33]. An acquisition time of 70,000 s was used and (i) the 295.21 and 351.92 keV ^214^Pb and 1120.29 keV ^214^Bi gamma-ray lines were selected to quantify the ^226^Ra activity concentration; (ii) the ^232^Th activity concentration was determined by using the 911.21 and 968.97 keV ^228^Ac gamma-ray lines; (iii) for ^40^K, the evaluation was carried out from its gamma-ray line at 1460.8 keV [34,35]. No true coincidence summing (TCS) correction was carried out for the 911.21 keV ^228^Ac gamma-ray line, taking into account that the ^212^Pb/^228^Ac ratio is very close to 1 [36].

Gamma spectrometry analysis were carried out through an electrically-cooled Ortec (Oak Ridge, TN, USA) HPGe detector positioned within lead wells to shield the contribution of background radioactivity [37], i.e., a direct biased semiconductor with 1.85 keV FWHM resolution, 40% relative efficiency and 64:1 peak to Compton ratio. The Eckert and Zigler (Berlin, Germany) Nuclitec GmgH traceable multi-nuclide radioactive standard, number BC4464, covering the energy range 59.54 keV–1836.09 keV, was employed to perform efficiency and energy calibrations [38]. The exact geometry of the sample was replicated by this calibration standard in an epoxy resin–matrix water equivalent. Ortec Gamma Vision software version 8.1 was used for efficiency transfer calculation and to collect and evaluate experimental data [39].

The activity concentration (Bq kg^−1^ dry weight, d.w.) of every identified radionuclide was calculated as follows [40]:

(1) $$C\left(Bq\;{kg}^{-1}\;d.w.\right)=\frac{N_{E}}{\varepsilon_{E}t\gamma_{d}M}$$

where N_E_ is the net area, ε_E_ is the efficiency for energy E and γ_d_ is the decay probability of the gamma photon; M is the dry mass of the sample (kg) and t is the acquisition time (s). For the assessment of the combined standard measurement uncertainty at coverage factor k = 2, the counting statistics, nuclear data library [41], calibration efficiency, sample quantity, and self-absorption correction, were considered.

Finally, the Italian Accreditation Body (ACCREDIA) recognized the high quality of the gamma spectrometry experiment results [42].

### 3.3. Radiological Health Risk

#### 3.3.1. Absorbed Gamma Dose Rate

The radiological health risk was first quantified through the absorbed gamma dose rate, D (nGy h^−1^), for indoor external exposure, calculated on the basis of the standard room model as reported in [43]:

D = 0.92C_Ra_ + 1.1C_Th_ + 0.08C_K_ (2)

where C_Ra_, C_Th_, and C_K_ are the average specific activities (the mean value of the five analyzed aliquots) of ^226^Ra, ^232^Th, and ^40^K in the analyzed specimen, respectively.

#### 3.3.2. Annual Effective Dose Equivalent

The following formula was used to determine an individual’s annual effective dose equivalent, AEDE (mSv y^−1^), using an 80% employment factor for indoor exposure [44]:

AEDE = (D − 50) × 8760 h × 0.7 Sv Gy^−1^ × 0.8 × 10^−6^ (3)

where the average dose rate value of 50 nGy h^−1^ for the background was discounted [43]. This value must be lower than 1 mSv y^−1^ to ensure a negligible radiological hazard [45].

#### 3.3.3. Activity Concentration Index

The following activity concentration index (ACI) was defined by the European Commission for identifying whether a dose criterion is met [43]:

ACI = (C_Ra_/300 + C_Th_/200 + C_K_/3000) (4)

It is related to the reference level applicable to external exposure to gamma radiation emitted by building materials indoors (AEDE), in addition to outdoor exposure, i.e., 1 mSv y^−1^ [45]. As a result, this index could only be utilized as a screening tool to detect materials that may pose a risk when utilized in buildings. Those with I > 1 should be avoided since these values correspond to dose rates higher than 1 mSv y^−1^.

#### 3.3.4. Alpha Index

The alpha index was calculated as follows [46]:

I_α_ = C_Ra_/200 (5)

In particular, it furnishes a measure of the exposure, originating from the indoor radon released by building materials, to the alpha radiation. In particular, to ensure a low exposure to indoor radon specific activity (<200 Bq m^−3^), the activity concentration of ^226^Ra must be less than 200 Bq kg^−1^. As a consequence, to guarantee a negligible risk related to radiation exposure, I_α_ must be less than 1.

### 3.4. ^222^Rn Exhalation Rate

In order to assess the radon exhalation rate, Mi.am (Piacenza, Italy) E-PERM electret ion chambers were employed to determine the ^222^Rn activity gathered in a vessel upon a specified build-up time [47], i.e., the “accumulator method” [48].

**Figure 2:**
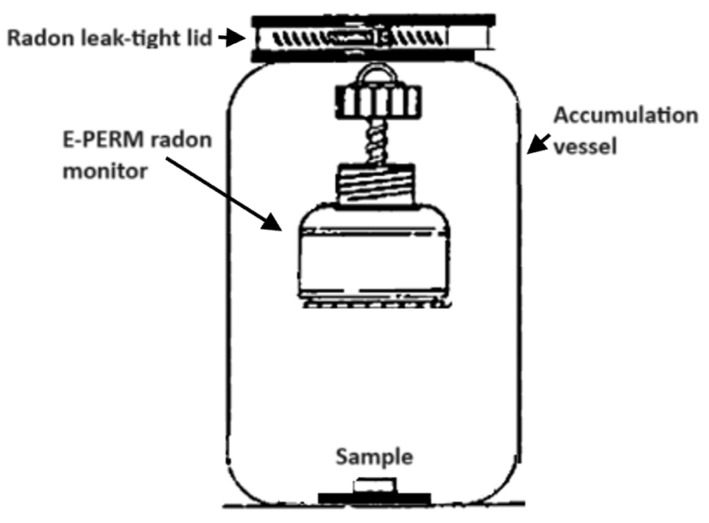
A schematic drawing of the experimental setup.

A schematic drawing of the experimental setup is shown in Figure 2.

The behavior of ^222^Rn specific activity, C_Rn_ (Bq m^−3^), vs. t, in a closed accumulation vessel can be written as follows:

(6) $$C_{Rn}=\frac{E(1-e^{-\lambda_{Rn}t})m}{V_{A}\lambda_{Rn}}+C_{Rn}^{0}e^{-\lambda_{Rn}t}$$

with E corresponding to the specific exhalation rate (Bq kg^−1^ h^−1^) from the sample, λ_Rn_ represents the decay constant associated to ^222^Rn (h^−1^), V_A_ is the accumulation vessel volume (m^3^), m is the sample mass (kg), and C^0^_Rn_ (Bq m^−3^) defines the ^222^Rn activity concentration corresponding to the accumulation time start (t = 0).

A glass jar with a screw cover and gasket was used to perform the accumulation measurements. It should be noted that it prevents radon losses and therefore it is not necessary to determine the loss factor of the vessel [49].

The E-PERM system relies on the ions produced by radon decay reducing the electrical potential of an electrostatically charged Teflon disk contained within a chamber Teflon disk contained inside a chamber. A combined measurement using a 210 mL chamber set up with a short-term electret was used to calculate the concentration of radon. In particular, radon activity concentration, C_Rn_ (Bq m^−3^), was quantified according to the following relationship:

(7) $$C_{Rn}=\frac{\left(V_{i}-V_{f}\right)}{C_{F}T}-BG$$

with V_i_ and V_f_ the measured initial and final electret voltages, respectively; C_F_ the calibration factor (V per Bq m^3^ d); T the exposure interval (days); and BG the background-related equivalent radon concentration of natural gamma radiation.

For the calibration factor, the following relationship was used:

(8) $$C_{F}=0.04589+0.0000155\frac{\left(V_{i}+V_{f}\right)}{2}$$

According to [50], the total accumulation volume for the E-PERM chamber after subtracting the excluded volume is 3.8 L. Hence, the “back diffusion” effect had no bearing on radon exhalation rate measurement because it was performed on a sample whose volume was more than ten times less than the container’s volume [51]. One by one, the sample and the electret ion chamber were inserted inside the jar; the sample was put on the bottom, and the chamber was suspended from the screw cap by a hook. After that, a unique rubber collar clamped with metal bands sealed the jar lid. The sample was kept for about twelve days in the container. At the end of the exposure time, after removing the electret ion chamber, an electrical potential drop was observed.

According to [46], the specific exhalation rate, E (Bq h^−1^ kg^−1^), was determined by the following:

(9) $$E=\frac{(C_{Rn}V_{A}\lambda_{Rn})/m}{1-(1-e^{-\lambda_{Rn}t})/\lambda_{Rn}T}$$

considering the mean concentration over the accumulation time, C_Rn_, of the sample. In our case, the net ^222^Rn concentration was evaluated by subtracting the blank reading, determined by introducing E-PERM chambers into the empty jar used for the experiments, exposed for the same time that was used for the experiment, from the total ^222^Rn concentration.

### 3.5. Inductively Coupled Plasma Mass Spectrometry (ICP-MS)

For the ICP-MS analysis, ~0.5–1.0 g of each aliquot of the analyzed sample, together with 3 mL of ultrapure (for trace analysis) HNO_3_ (67–69%) and 9 mL of ultrapure (for trace analysis) HCl (32–35%) (aqua regia), were directly introduced into a 100 mL TFM vessel. Acid digestion was performed using a Milestone microwave unit system (Milestone, Bergamo, Italy), Ethos touch control, in three steps: 15 min at 1000 W and 200 °C; 10 min at 700 W and 200 °C; 10 min cooling [52]. This microwave extraction method is designed to mimic extraction using conventional heating with nitric acid (HNO_3_), or alternatively, nitric acid and hydrochloric acid (HCl), according to [53], without the total decomposition of the sample.

For the measurement, a Thermo Scientific (Waltham, MA, USA) iCAP Qc ICP-MS was employed [54]. The sample introduction system consisted of a Peltier cooled (3 °C), baffled cyclonic spray chamber, PFA nebulizer and quartz torch with a 2.5 mm, i.e., removable quartz injector. The instrument was operated in a single collision cell mode, with kinetic energy discrimination (KED), using as collision gas pure Helium. The sample was analyzed using a Cetac ASX-520 (Teledyne Cetac Technologies, Omaha, NE, USA). Detected metals were Sb, As, Cd, Co, Hg, Ni, Pb, Cu, Tl, V and Zn. U, Th and K were not detected for lack of calibration standards. Noteworthy, the quality of the ICP-MS experimental results was certified by ACCREDIA [42].

### 3.6. Level of Metals Contamination

The level of metal contamination in the analyzed sample was evaluated by calculating the geo-accumulation index (I_geo_). This is given as follows [55]:

(10) $$I_{geo}={Log}_{2}\left[C_{n}/\left(kB_{n}\right)\right]$$

where C_n_ is the average concentration of the potentially hazardous trace element in the sample, B_n_ is the geochemical background value in average shale [56] of the element n and k = 1.5 is the background matrix correction factor that was introduced to account for possible deviations in the background readings due to lithogenic effects [56].

### 3.7. X-ray Diffraction

X-ray diffraction measurements were carried out through a Malvern PANalytical (Malvern, UK) Empyrean Diffractometer with Cu Kα radiation on a Bragg–Brentano theta–theta goniometer, equipped with a solid-state detector, PIXcel [57].

To perform the XRD investigation, approximately 1 g of finely powdered granodiorite was employed for each aliquot of the investigated sample. The measurement conditions of the X-ray diffraction analysis were set at 40 kV and 40 mA, while the XRD pattern was recorded within the 2θ angle range from 2° to 70°, with a step size of 0.053° and a scan step time of 4.08 s. Concerning the Cu Kα2 component of the raw data and background correction, two different adjustments were applied; respectively, the first one was removed using Highscore Plus software version 5.1, whereas the second one was reduced using a digital filter. Instead, with the aim of identifying the mineral components present in the considered granodiorite powdered, the observed peak positions were compared with the ICDD JCPDS database [58].

### 3.8. µ-Raman Scattering

µ-Raman scattering measurements were carried out using a HORIBA Scientific (Kyoto, Japan) LabRAM HR Evolution Raman spectrometer, coupled with an integrated Microscope central (Feasterville-Trevose, PA, USA) Olympus BX41 microscope. The excitation wavelength was of 532 nm (2.33 eV). The laser beam was focused on the sample surface using a Microscope central (Feasterville-Trevose, PA, USA) Olympus 50XLF (long focal length) objective, resulting in a spot size of approximately 2 μm. Low laser power (below 1 mW) was used to minimize sample heating and possible damage. The spectral resolution was 0.2 cm^−1^. Measurements were collected on a minimum of ten distinct grains of each aliquot of the investigated sample [59].

## 4. Results and Discussion

### 4.1. Radioactivity and Radon Exhalation Analysis

The average activity concentrations of ^226^Ra, ^232^Th, and ^40^K in the investigated rock, together with the average specific ^222^Rn exhalation rate (the mean value for the five aliquots of the investigated rock), are reported in Table 1.

It is worthy of note that, in the investigated sample, (i) the activity concentration of ^226^Ra is lower than the average world value for this environmental matrix, i.e., 35 Bq kg^−1^; (ii) the activity concentration of ^232^Th is a little bit higher than the average world value, i.e., 30 Bq kg^−1^; (iii) the activity concentration of ^40^K is significantly higher than the average worldwide one, i.e., 400 Bq kg^−1^ [1]. In particular, this anomalous behavior for ^40^K may be due to feldspars in the area or the granite itself, and for this reason it deserves a more critical interpretation based on the specific granodiorite mineralogical composition, that will be given in the following. Moreover, the average specific ^222^Rn exhalation rate was found to be (0.0040 ± 0.0009) (Bq h^−1^ kg^−1^). As widely reported in the literature, magmatic stones (such as granodiorite) can be considered as major sources of radon emanation [60]. Nevertheless, the radon exhalation rate from magmatic rock samples can show wide dispersion with values spread over two orders of magnitude. Noteworthy, our mean value is in good agreement with the corresponding ones that were measured worldwide [61,62,63].

### 4.2. Radiological Hazard Effects Assessment

The absorbed gamma dose rate (D), the annual effective dose equivalent (AEDE), the activity concentration index (ACI) and the alpha index (I_α_), as assessed through Equations (2)–(5), are reported in Table 2.

As reported in the literature, the absorbed gamma dose rate for the investigated rock sample is ascribable to the lithologic component of the sampling site [64]. Furthermore, the annual effective dose equivalent due to the activities of ^226^Ra, ^232^Th, and ^40^K in the investigated sample was found to be 448 µSv y^−1^, lower than the threshold value set to 1 mSv y^−1^ [45]. Next, the investigated granodiorite’s usefulness as a component in construction materials was determined by evaluating its activity concentration index. It was found to be 0.6, less than unity, thus indicating negligible radiological risks associated with exposure to gamma radiation. Finally, concerning the alpha index value, it was found to be 0.08, still in this case less than 1 and thus preventing exposure to indoor radon concentrations above 200 Bq m^−3^.

### 4.3. Metals’ Analysis

Table 3 reports the average metal content (µg g^−1^ d.w.) of the rock under examination, as determined by ICP-MS analysis.

In particular, the resulting concentrations for all the detected elements were found to be less than the contamination threshold values provided by [65] (reported in Table 1). Accordingly, such concentrations cannot be regarded as effective pollutants as far as the natural metallic concentrations related to the investigated rock are concerned. Therefore, they have no unintended consequences and do not endanger environmental wellbeing. Consequently, they do not pose a harm to human health.

### 4.4. Estimation of the Level of Metal Contamination

Table 4 reports I_geo_ values for the investigated metals of the analyzed granodiorite.

They were interpreted according to [66]. In our case, all values are < 0, indicating no contamination for the detected metals.

### 4.5. XRD Analysis

**Figure 3:**
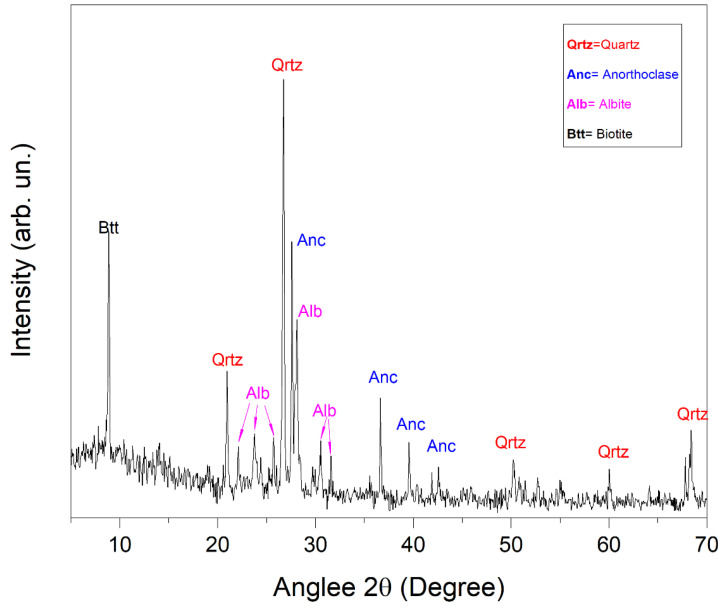
X-ray diffraction spectrum of the granodiorite powder. The peaks correspond to the diffraction angles (2θ) and their intensities, indicating the crystallographic planes within the sample. The positions and intensities of these peaks reveal the presence of quartz, anorthoclase, albite, and biotite, highlighting the mineralogical composition of the sample.

The most representative X-ray diffraction spectrum is shown in Figure 3.

By comparing the measured diffraction peak positions with the ICCD and RRUFF databases, the minerals were identified. In particular, XRD analysis revealed that the sample was characterized by the presence of quartz (SiO_2_, Ref. COD: 96-101-1177), anorthoclase ((Na,K)AlSi_3_O_8_, Ref. COD: 96-900-0858), albite (Na(AlSi_3_O_8_), Ref. COD: 96-900-1634) and biotite ((H,K)2(Mg,Fe)2(Al,Fe)2(SiO_4_)3, Ref. COD: 96-900-0026). This mineralogical composition of the granodiorite can explain the observed natural radioactivity content described in the previous sections. In particular, anorthoclase, a potassium-rich feldspar, and biotite, a member of the mica group, both contain significant amounts of potassium, which may explain the high value of the ^40^K activity concentration reported in Table 1 [67]. Moreover, during the crystallization of granodiorites from molten magma, uranium and thorium can be incorporated into the forming phases, replacing the ions of other elements present in the crystal structure. This process, known as “isomorphous substitution”, can lead to granodiorites containing concentrations of uranium and thorium within their constituent mineralogical phases [68]. In addition, the Th/U ratio can be influenced by various geological and petrogenetic processes. Generally, thorium and uranium behave differently during magmatic and metamorphic processes. Th tends to be less mobile compared to U during hydrothermal metamorphism and alteration processes, leading to a relative enrichment of Th over U in certain rocks. Additionally, the Th/U ratio is often used as an indicator to understand the magmatic fractionation processes and magma provenance. In our case, the high Th/U ratio observed in the granodiorite sample might be attributed to the presence of Th-rich accessory minerals [69,70].

### 4.6. µ-Raman Analysis

**Figure 4:**
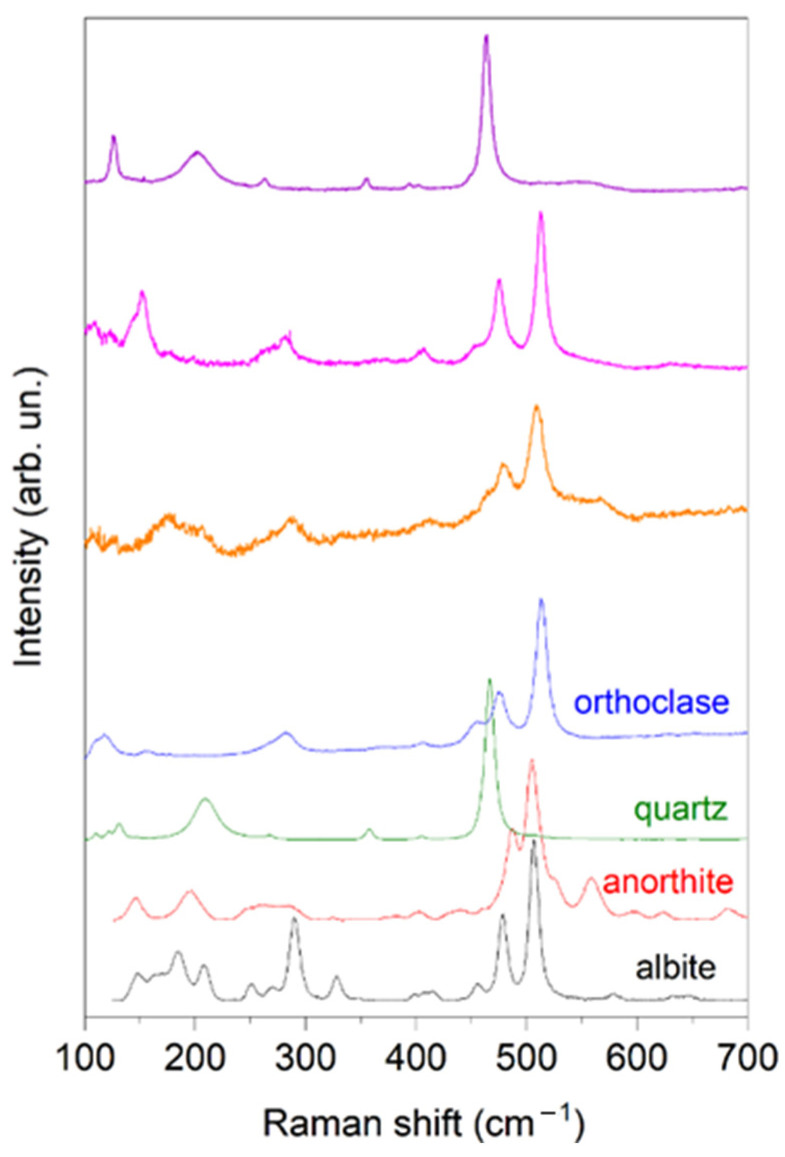
Raman spectra collected at various positions on several grains of the powdered sample. The identified peaks correspond to minerals such as quartz, albite, anorthite, and orthoclase, and their assignment provides information on the mineralogical and chemical properties of the powdered granodiorite. Reference spectra of the most abundant species, as determined by XRD analysis, are provided for comparison.

To investigate the mineral components, in terms of the crystalline microstructure, µ-Raman spectra were collected at different spots of several grains in the sample. Figure 4 shows three of the most representative micro-Raman spectra acquired in the analyzed sample.

As a result, quartz (RRUFF ID: R040031) was clearly identified, together with orthoclase (RRUFF ID: R040055), anorthite (RRUFF ID: R040059) and albite (RRUFF ID: R040068). These minerals were chosen due to their higher abundance, as determined by XRD analysis. Finally, the selection of anorthite and orthoclase minerals was made due to the absence of anorthoclase in the RRUFF library; however, both the aforementioned minerals (anorthite and orthoclase) are contained in the anorthoclase mineral, and this makes their analysis meaningful. Such results validate the XRD analysis.

## 5. Conclusions

The natural radioactivity content, radon exhalation and metal pollution of a granodiorite rock sample from Stilo, in the Calabria region, Southern Italy, were investigated through HPGe gamma-ray spectrometry, E-PERM electret ion chambers and ICP-MS, respectively, and reported as a case study. The evaluation of the absorbed gamma dose rate, the annual effective dose equivalent, the activity concentration index and the alpha index were carried out in order to evaluate any possible radiological hazard related to radiation exposure from the analyzed rock. In particular, the obtained values of AEDE turned out to be lower than the action levels provided by the Italian legislation for the general population, thus confirming no hazard effects from the radiological point of view. In addition, I and I_α_ were found to be lower than unity, indicating negligible radiological risks associated with exposure to gamma radiation and unlikely exposure to indoor radon concentrations above 200 Bq m^−3^.

Going on, the metal-related ecological hazard to the ecosystem was evaluated through the quantification of the geo-accumulation index for Sb, As, Cd, Co, Hg, Ni, Pb, Cu, Tl, V and Zn in the investigated rock. In particular, the obtained I_geo_ values indicated no contamination by metals.

Finally, XRD and µ-Raman spectroscopy were used to properly correlate the radioactivity content with the mineralogical and geochemical composition of the investigated sample, thus recognizing the presence of anorthoclase and biotite as the main factors responsible for the increased natural radioactivity concentration observed in the investigated rock.

## Figures and Tables

**Table 1:** The average activity concentrations of ^226^Ra, ^232^Th, and ^40^K, in the investigated rock, together with the average specific ^222^Rn exhalation rate.

C_Ra_ (Bq kg^−1^ d.w.)	C_Th_ (Bq kg^−1^ d.w.)	C_K_ (Bq kg^−1^ d.w.)	^222^Rn Exhalation Rate (Bq h^−1^ kg^−1^)
15.7 ± 2.3	48.5 ± 7.6	918 ± 129	0.0040 ± 0.0009

**Table 2:** The absorbed gamma dose rate (D), the annual effective dose equivalent (AEDE), the activity concentration index (ACI) and the alpha index (Iα) for the investigated sample.

D (nGy h^−1^)	AEDE (µSv y^−1^)	I	I_α_
141	448	0.6	0.08

**Table 3:** The average metal content (µg g^−1^ d.w.) for the analyzed sample, as obtained through ICP-MS analysis.

ICP-MS Analysis		
Metal	µg g^−1^ d.w.	Threshold Limit
Sb	0.13	10
As	1.67	20
Cd	0.04	2
Co	4.91	20
Hg	0.10	1
Ni	4.17	120
Pb	5.29	100
Cu	4.41	120
Tl	0.60	1
V	27.9	90
Zn	61.3	150

**Table 4:** Calculated values of the geo-accumulation index (I_geo_) for all detected metals.

	I_geo_
Sb	−4.11
As	−3.55
Cd	−3.49
Co	−2.54
Hg	−2.58
Ni	−4.61
Pb	−2.50
Cu	−3.94
Tl	−1.81
V	−2.81
Zn	−1.22

## Data Availability

The original contributions presented in the study are included in the article, further inquiries can be directed to the corresponding author.
